# Assay for interferon gamma release as a novel marker in pediatric patients with systemic lupus erythematosus

**DOI:** 10.1186/s12969-024-01008-9

**Published:** 2024-08-01

**Authors:** Song Zhang, Xue Li, Huishan Chen, Xianfei Gao, Zhe Cai, Huasong Zeng

**Affiliations:** 1 Department of Allergy, Immunology and Rheumatology, Guangzhou Women and Children’s Medical Center, Guangzhou Medical University，Guangdong Provincial Clinical Research Center for Child Health, Guangzhou, 510623 China; 2 Department of Rheumatology and Immunology, Guangdong Provincial Key Laboratory of Major Obstetric Diseases, Guangdong Provincial Clinical Research Center for Obstetrics and Gynecology, The Third Affiliated Hospital, Guangzhou Medical University, Guangzhou, 510623 China; 3 Guangzhou Institute of Pediatrics, Guangzhou Women and Children’s Medical Center, Guangzhou Medical University, Guangzhou, 510623 China

**Keywords:** Systemic lupus erythematosus, Diagnostic biomarker, *Mycobacterium tuberculosis* IFN-γ release assay, SLE disease activity index

## Abstract

**Background:**

The interferon-gamma (IFN-γ) release assay (IGRA) is an important laboratory diagnosis for latent *Mycobacterium tuberculosis* (TB) infection. The TB-IGRA measures the release of IFN-γ from peripheral blood cells, who are exposed to TB antigen (Ag), mitogen (MT), or negative/nil control (NL) in vitro. While, an exceptional higher TB Ag-NL level will reflect an elevation of peripheral lymphocytes released IFN-γ in a same condition. Therefore, we found that the elevated levels of TB Ag-NL could become a new biomarker for the diagnosis and treatment of pediatric systemic lupus erythematosus (SLE) patients.

**Methods:**

We have analyzed the clinical data of 776 children who are underwent TB-IGRA testing in the Department of Allergy and Rheumatology of Guangzhou Women and Children’s Medical Center from 2018 to 2020. To investigate the association between TB Ag-NL and SLE, we have analyzed the clinical data of 47 SLE patients and TB Ag-NL testing results, and then evaluated the association between TB Ag-NL and SLE disease activity.

**Results:**

The TB Ag-NL levels were significantly higher in patients with active SLE than those in inactive SLE (*p* = 0.0002). The TB Ag-NL levels were positively correlated with the SLE disease activity index (SLEDAI) and laboratory diagnosis parameters. The mean value of TB Ag-NL in SLE patients (0.04191 ± 0.07955, IU/mL) were significantly higher than those in patients with juvenile dermatomyositis (JDM) (0.0158 ± 0.0337, IU/mL, *p* = 0.036), juvenile idiopathic arthritis (JIA) (0.0162 ± 0.0388, IU/mL, *p* = 0.001), and healthy controls (HC) (0.0001 ± 0.0027, IU/mL, *p* = 0.0003). Therefore, the elevated TB Ag-NL levels could serve as a potential diagnostic biomarker of SLE, especially for the active SLE.

**Conclusion:**

The detection of IFN-γ release levels by the TB-IGRA may be useful to assess SLE disease activity in pediatric patients with active SLE.

**Supplementary Information:**

The online version contains supplementary material available at 10.1186/s12969-024-01008-9.

## Key messages

Spontaneous IFN-γ release is associated with Systemic lupus erythematosus in children.

IFN-γ-releasing potential, as measured by the mycobacterium tuberculosis IFN-c release assay, associates with Systemic lupus erythematosus activity in children .

IFN-γ release assays may offer a novel, blood-based approach to assessing SLE disease activity in children.

**Supplementary Information:**

The online version contains supplementary material available at 10.1186/s12969-024-01008-9.

## Introduction

Systemic lupus erythematosus (SLE) is a chronic autoimmune disease which is characterized by the secretion of autoantibodies and generation of cellular antigens, tissue inflammation and organ damages [1]. Besides the antibody production, B cells can interact with T cell antigens and secrete pro-inflammatory cytokines and chemokines, leading to inflammatory reactions that promote disease development [2–4]. Among the cytokines involved in SLE pathogenesis, type I interferon (IFN) plays an important role. The increase serum IFNα levels or IFN-induced gene expressions which usually associated with disease activity and clinical manifestation are found in most SLE patients. The IFN-γ, produced by T helper cells, cytotoxic T cells, and natural killer cells [5], plays an important role in innate immunity and acquired cell-mediated immunity. The IFN-γ overproduction is also found in SLE patients [6]. IFN-γ over activation promotes a chronic pro-inflammatory cascade that leads to the increased organ damage in SLE [7]. Upregulated IFN-γ activity is hypothesized to enhance antigen presentation and promotes the accumulation of pathogenic autoantibodies, which is leading to the IFN-α signaling dysregulation and increasing the likelihood of preclinical progression to clinical SLE [8].

The *Mycobacterium tuberculosis* (TB) IFN-γ release assay (TB-IGRA) is generally used to assess the likelihood of TB infection in clinical trials. The third-generation QuantiFERON-TB Gold (QFT) In-Tube IGRA, a whole-blood IFN-γ release assay, is widely used to assess the possibility of TB infection in clinical trials. The test relies on the release of IFN-γ from the memory T-lymphocytes, when they are exposed to the TB antigens. The IFN-γ release is measured after the incubation (18–24 h) with three different stimuli like (i) TB antigen (Ag); (ii) mitogen (MT) and (iii) a negative/nil condition (NL). IGRA-MT is proposed to assess the IFN-γ release capacity of peripheral blood cells with mitogen stimulation; and (iii) IGRA-NL aims to measure the background of IFN-γ release in each patient. In this report, we have analyzed the assay results from QFT In-Tube IGRA detection. The difference between these values is used to calculate the likelihood of TB infection. Recently, the IFN-γ release has been reported to assess immunity in infants with congenital cytomegalovirus infection and activity in adult SLE [7–9]. In this study, we used the TB-IGRA to analyze the IFN-γ release in 776 hospitalized children to further clarify its correlation between SLE characteristics and clinical diagnostic parameters. Here, we found the IFN-γ release played an important role on reflecting the organ damages in the pediatric patients with active SLE. Therefore, we hypothesized that the IFN-γ release is associated with the disease activity of SLE.

## Methods

### TB-IGRA measurement

We have analyzed 776 sequential TB Ag-NL results which are obtained from the Guangzhou Women and Children’s Medical Center during 2018 to 2020. The number of TB-IGRA testing results of each patient are recorded according to the formula: TB Ag-NL = TB antigen-negative/nil control. Before the TB-IGRA examination, the patient was in the first visit and had not received any treatment. The information of each patient, including gender, age, symptoms, signs, and clinical diagnosis are also recorded accordingly.

### SLE subjects

We have identified all of patients with SLE/ANA+, who at least have one test of the TB-IGRA assay during 2018 to 2020. All clinical investigations were conducted in accordance with the guidelines of the Declaration of Helsinki and the Clinical Practice guidelines.All the subjects with SLE are met the Systemic Lupus Erythematosus International Collaborating Clinics (SLICC) criteria for SLE classification [10]. In this study, the SLE disease activity index (SLEDAI) and the TB-IGRA assay were matched with their clinical parameters and laboratory diagnosis acquired on the same day. The active SLE patients are defined as SLEDAI score > 4 [11]. While, other subjects who’s SLEDAI score ≤ 4 are defined as the inactive SLE. In addition, to exclude the impact of TB caused IFN-γ release, there are 47 eligible pediatric patients with SLE have enrolled for further analysis. Interferon gamma release were negative, and tuberculosis infection was excluded in all 47 patients with systemic lupus erythematosus。.

### Statistical analysis

Statistical analysis of clinical data was described in the section of each assay. Results were expressed as mean ± standard deviation (SD) or median with interquartile range (IQR, 25-75%) for normally distributed data. Mann-Whitney U-tests were tested the continuous variables. All hypotheses were two-tailed. The probability (P)-values < 0.05 were considered significant. The receiver operating characteristic (ROC) curve, based on the results of logistic regression analysis by GraphPad Prism software (version 8.3.0 for Windows; GraphPad Software, La Jolla, CA, USA), was used to evaluate the diagnostic value of IFN-γ release in the progression of SLE. Spearman’s rank correlation test was used to evaluate the lineal correlations between TB Ag-NL with clinical parameters of pediatric SLE patients. r = correlation coefficient. Data were analyzed using GraphPad.

## Results

### Baseline characteristics of the study subjects

Among the 47 patients acquired in this study, 40 of them were female patients (85.1%). There were 32 cases of active SLE (68.1%) and 15 cases of inactive SLE (31.9%). The mean ± SD value of age was 11.53 ± 1.94 years. The mean ± SD value of SLE duration was 1.85 ± 2.21 years. The mean ± SD value of SLEDAI score was 10.89 ± 9.73 years. These data were summarized in Table 1.

**Table 1:** Clinical features of the SLE subjects, median (IQR)

	SLE subjects, *N* = 47	TB Ag-NL ^low^, *N* = 34	TB Ag-NL ^high^, *N* = 13	*p*-value
Baseline characteristics				
Age (year)	12 (10–13)	12 (11–13)	11 (9–12)	0.0603
Gender (Male/Female), n (%)	7/40 (17.50)	4/30 (13.33)	3/10 (30)	0.3769
Disease duration since diagnosis (year)	1 (0.1-3)	2 (0.6-3)	0.3 (0.1–0.9)	**0.0273**
Clinical features of SLE subjects				
SLEDAI ≤ 4 (*N* = 15)	2 (2–2)	2 (2–2)	0 (0–0)	**< 0.0001**
SLEDAI > 4 (*N* = 32)	14 (8-17.50)	9 (7.50–14)	20 (15-28.50)	**< 0.0001**
SLICC features, n (%)				
Acute cutaneous lupus	16/47 (34.04)	8/34 (23.53)	8/13 (61.54)	**0.0198**
Chronic cutaneous lupus	4/47 (8.51)	3/34 (8.82)	1/13 (7.69)	1.000
Neurologic disorder	2/47 (2.13)	0/34 (0)	2/13 (15.38)	0.0722
Oral/nasal ulcers	4/47 (8.51)	2/34 (5.88)	2/13 (15.38)	0.5723
Joint disease	6/47 (12.77)	1/34 (2.94)	5/13 (38.46)	**0.0042**
Serositis	5/47 (10.64)	0/34 (0)	5/13 (38.46)	**0.0008**
Hemolytic anemia	11/47 (23.40)	4/34 (11.76)	7/13 (53.85)	**0.005**
Leukopenia	7/47 (14.89)	3/34 (8.82)	4/13 (30.77)	0.1723
Thrombocytopenia	3/47 (6.38)	1/34 (2.94)	2/13 (15.38)	0.0503
Antiphospholipid syndrome	2/47 (2.13)	2/34 (5.88)	0/13 (0)	1.000
ANA	45/47 (95.74)	33/34 (97.06)	12/13 (92.31)	0.0774
Anti-dsDNA	45/47 (95.74)	33/34 (97.06)	12/13 (92.31)	**0.0003**
Low complement	28/47 (59.57)	17/34 (50)	11/13 (84.62)	**0.0463**
Anti-Smith	7/47 (14.89)	5/34 (14.71)	2/13 (15.38)	1.000
Renal involvemen	21/47(44.68)	10/34(29.41)	11/13(84.62)	**0.0009**

*P*-values in bold font represent the statistically significant (*P <* 0.05). IQR, interquartile range (25-75%) of the data. TB Ag-NL ^low^ or TB Ag-NL ^high^ is the value of TB Ag-NL lower or higher than the mean value of TB Ag-NL, respectively. SLICC, systemic lupus erythematosus international collaborating clinics. ANA, antinuclear antibodies. Anti-dsDNA, anti-double stranded DNA. N, number

**Fig. 1:**
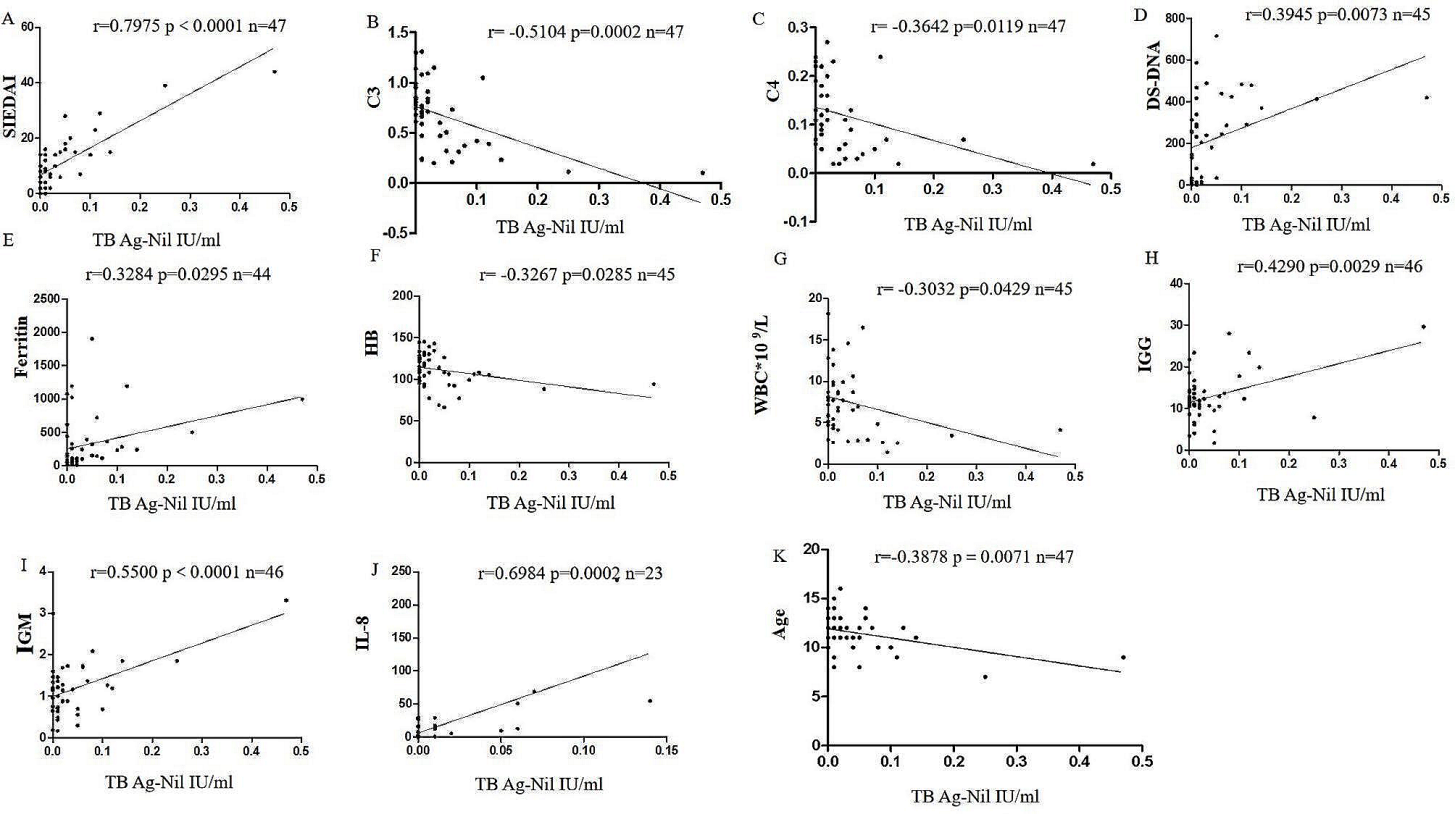
Correlationship between TB Ag-NL levels and systemic lupus erythematosus (SLE) clinical features. (**A**) Correlationship between TB Ag-NL levels and SLEDAI scores. (**B**) Correlationship between TB Ag-NL levels and C3. (**C**) Correlationship between TB Ag-NL levels and C4. (**D**) Correlationship between TB Ag-NL levels and anti-dsDNA. (**E**) Correlationship between TB Ag-NL levels and Ferritin. (**F**) Correlationship between TB Ag-NL levels and Hemoglobin (HB). (**G**) Correlationship between TB Ag-NL levels and White blood cell (WBC). (**H**) Correlationship between TB Ag-NL levels and IgG. (**I**) Correlationship between TB Ag-NL levels and IgM. (**J**) Correlationship between TB Ag-NL levels and IL-8. (**K**) Correlationship between TBAg-NL levels and Age

Thomason, JL et al. demonstrated that a similar relationship between IGRA-NL/MT and SLEDAI was found in adult patients [12]. Indeed, we have observed a stronger correlationship between TB Ag-NL and SLEDAI (*r* = 0.7975, *P <* 0.0001, *n* = 47) (Fig. 1A) in our study. We also observed a stronger negative correlations between TB Ag-NL and the levels of complement-3 (C3) (*r* = -0.5104, *p* = 0.0002, *n* = 47) (Fig. 1B) and C4 (*r* = -0.3642, *p* = 0.0119, *n* = 47) (Fig. 1C). The related laboratory parameters of patients were also studied when testing TB-IGRA. The TB Ag-NL levels were also correlated with the anti-dsDNA (*r* = 0.3945, *p* = 0.0073, *n* = 45) (Fig. 1D). Previous reports found that IFN-γ could down-regulate the level of intracellular ferritin and increase serum ferritin [13–15]. We also observed the positive correlations between TB Ag-NL with serum ferritin levels (*r* = 0.3284, *p* = 0.0295, *n* = 44) (Fig. 1E), IgG (*r* = 0.4290, *p* = 0.0029, *n* = 46) (Fig. 1H), and IgM (*r* = 0.5500, *P <* 0.0001, *n* = 46) (Fig. 1I); and negative correlations with Hemoglobin (HB) (*r* = -0.3267, *p* = 0.0285, *n* = 45) (Fig. 1F) and White blood cell (WBC) (*r* = -0.3032, *p* = 0.0429, *n* = 45) (Fig. 1G). Moreover, the levels of serum interleukin (IL)-8 were reported to be associated with severe SLE nephritis and neuropsychiatric SLE [16, 17]. We also observed a strong positive association between TB Ag-NL and IL-8 levels in SLE (*r* = 0.6984, *p* = 0.0002, *n* = 23) (Fig. 1J).

### Comparison of TB Ag-NL levels in patients with SLE, JIA, and JDM and healthy controls (HC)

**Fig. 2:**
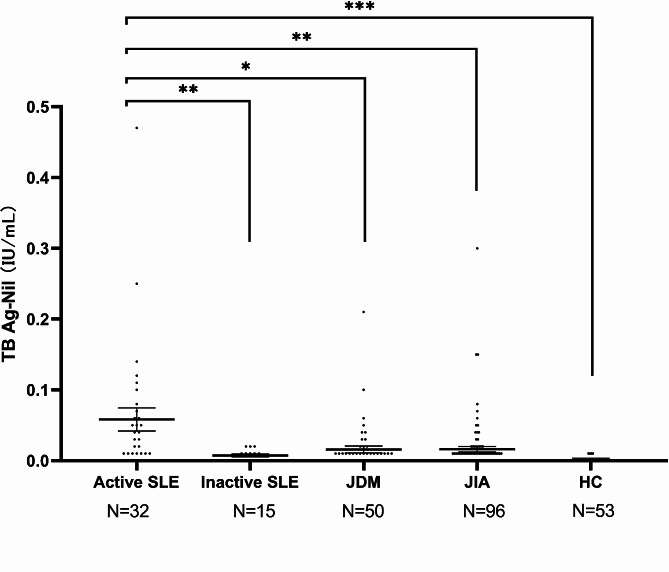
The differences of TB Ag-NL levels between patients with SLE (*N* = 47), juvenile dermatomyositis (JDM, *N* = 50), or juvenile idiopathic arthritis (JIA, *N* = 96), and healthy controls (HC, *N* = 53). Data are expressed as medians, and the error bars indicate interquartile ranges. *P <* 0.05 represents the statistically significant

To evaluate whether the TB Ag-NL level is a novel latent biomarker for diagnosing SLE, we compared the TB Ag-NL levels between pediatric SLE patients with juvenile idiopathic arthritis (JIA), juvenile dermatomyositis (JDM) and HC. TB-IGRAL levels were measured in 50 children with dermatomyositis, 96 with juvenile idiopathic arthritis and 53 normal controls. The mean value of TB Ag-NL in SLE patients (0.04191 ± 0.07955, IU/mL) were significantly higher than those in patients with JDM (0.0158 ± 0.0337, IU/mL, *p* = 0.036), JIA (0.0162 ± 0.0388, IU/mL, *p* = 0.001), and HC (0.0001 ± 0.0027, IU/mL, *p* = 0.0003) (Fig. 2).

### Diagnostic values of TB Ag-NL levels in pediatric patients with SLE

**Fig. 3:**
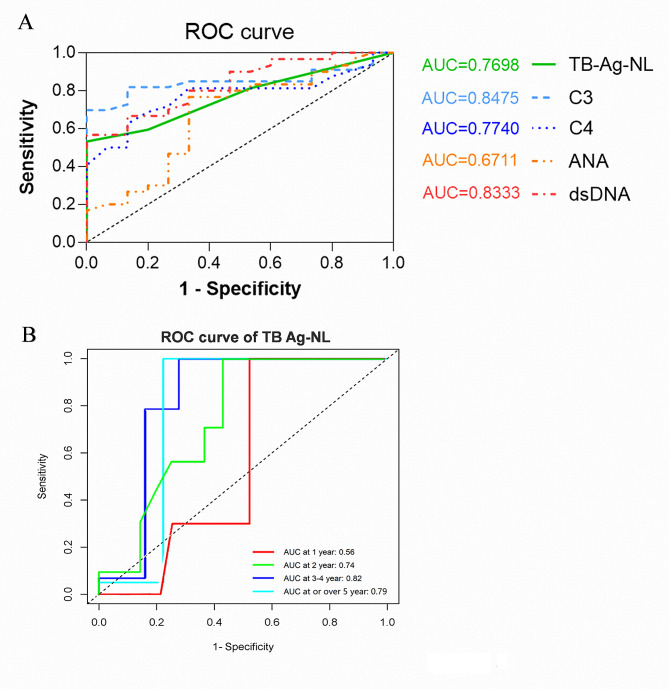
ROC curve analysis of TB Ag-NL levels in pediatric SLE. (**A**) The area under the ROC curve (AUC) of TB Ag-NL, C3, C4, ANA and Anti-dsDNA are shown in corresponded colors. (**B**) The ROC curve and AUC value of TB Ag-NL with the duration of SLE disease. Different colors show the corresponded years at duration of SLE.

We compared the sensitivity and specificity of TB Ag-NL levels with traditional markers such as C3, C4, ANA, and anti-dsDNA in pediatric SLE patients. The area under the ROC curve (AUC) of TB Ag-NL was 0.7698 (95% confidence interval (CI): 0.6388, 0.9). The AUC of C3 was 0.8475 (95% CI: 0.7368, 0.9), which was slightly higher than C4 (0.7740, 95% CI: 0.6408, 0.9), ANA (0.6711, 95% CI: 0.4792, 0.8) and anti-dsDNA (0.8333, 95% CI: 0.7174, 0.9). Indeed, the AUC value of TB Ag-NL was superior to C4 and ANA, and was almost close to the values of anti-dsDNA and C3, which were commonly used to determine the activity of lupus (Fig. 3A). In addition, we also found that the AUC value of TB Ag-NL had a raising trend with the duration of disease (Fig. 3B). It may imply a diagnostic worth of TB Ag-NL for the potential pediatric patients with long term SLE disease.

## Discussion

TB infection should be excluded during the diagnosis and treatment of SLE in children. In this study, we provide evidence that high levels of IFN-γ release are associated with SLE disease activity in children by the TB-IGRA measurements, which is a routine clinical trial in our hospital. Among 47 SLE patients in this study who undergo the TB-IGRA testing, the SLICC features like acute cutaneous lupus, joint disease, serositis, hemolytic anemia, anti-dsDNA and low complement are significantly different between TB Ag-NL ^low^ and TB Ag-NL ^high^ groups (Table 1). This may suggest a potential association between IFN-γ release and SLE features among pediatric SLE patients, which means the higher levels of TB Ag-NL, the stronger correlations with SLEDAI, anti-dsDNA, total IgG, C3 and C4 (Fig. 1).

Thus, the IFN-γ release, which is measured by the widely used TB-IGRA measurement in clinics, may be a useful biomarker for pediatric SLE patients to distinguish from other rheumatoid disease such as JDM and JIA (Fig. 2), where is consistent with a report on adult SLE [12]. Several studies have demonstrated a positive correlation between the active SLE (especially nephritis) and the high IFN serum levels [16–20].

In some previous studies, the high levels of TB Ag-NL are correlated with the increased IL-8, which plays an important role in the development of lupus, skin damage, and nephritis [16, 21]. The canonical pro-inflammatory factor IL-8 may associate with the release of IFN-γ in SLE patients. In our study, the TB Ag-NL level is positively correlated with the level of serum IL-8 in pediatric SLE patients (shown in Fig. 2). In addition, the higher level of TB Ag-NL in SLE rather than those in JDM and JIA patients, as well as HC was consistent with previous report [22]. This suggests that the pathogenesis of lupus is related to the IFN pathway [23, 24]. The activation of IFN pathway served as a marker for more severe disease involving the kidneys, hematopoietic cells, and/or the central nervous system in SLE [20, 25–27]. Our observations have raised an important question regarding the limits of TB-IGRA applications in pediatric SLE. From the TB Ag-NL results, nearly a quarter of severe SLE patients are undetectable. It may be associated with T-cell failure, a nonfunctional state in which antigen persists [28, 29]. However, the mechanism of undetectable TB Ag-NL levels in severe SLE and whether it is associated with T-cell failure need to be further addressed in future.

## Conclusions

Summarily, in this study the elevated TB Ag-NL in active SLE was correlated with SLEDAI scores. This finding suggested that the elevated TB Ag-NL may be used as a biomarker for manifesting the disease activity in pediatric SLE patients. Furthermore, due to a fewer number of SLE patients enrolled in this study, some of the statistical results remain to be determined. It should pay more attention for interpreting the results of TB-IGRA in patients with active lupus. It is much better to assess lupus activity by combining TB Ag-NL with some classical SLE diagnostic parameters, such as C3, ANA and anti-dsDNA.

### Electronic supplementary material

Below is the link to the electronic supplementary material.

Supplementary Material 1

## Data Availability

Not applicable.

## Abbreviations

Ag: antigenAg
HC: Healthy controls
IGRA: Interferon-gamma (IFN-γ) release assay
JDM: Juvenile dermatomyositis
JIA: Juvenile idiopathic arthritis
MT: Mitogen negative/nil control
NL: Negative/nil contro
SLE: Systemic lupus erythematosus
SLEDAI: Disease activity index
SLICC: International Collaborating Clinics
TB: My cobacterium tuberculosis
QFT: QuantiFERON-TB Gold
