# Supplementary material for: Assay for interferon gamma release as a novel marker in pediatric patients with systemic lupus erythematosus
Source: Pediatr Rheumatol Online J. 2024 Aug 1;22:70. doi: 10.1186/s12969-024-01008-9 (PMC11292859; doi:10.1186/s12969-024-01008-9)
Supplement: Supplementary file 1 — Supplementary Material 1 [file 12969_2024_1008_MOESM1_ESM.doc]

Field Chief Editor

Dear Editor:

# I wish to submit a article for publication in Pediatric Rheumatology, titled “Assay for interferon gamma release as a novel marker in pediatric patients with systemic lupus erythematosus.” The paper was coauthored by Song Zhang ,Xue Li 。

This study investigated activation of IFN-γ release levels by TB-IGRA may be a useful tool for assessing disease activity in patients with mild to moderate SLE of children. In addition, T-cell spot of tuberculosis assay is more suitable than TB-IGRAL for the assessment of LTBI in severe active lupus.

# We believe that our study makes a significant contribution and will be of interest to the readership of your journal because there are few studies on the markers of systemic lupus erythematosus in children, which can predict the activity of the disease. Publication of the results of this study in Pediatric Rheumatology could serve to accelerate the research and clinical application of a new methodology.

This manuscript has not been published or presented elsewhere in part or in entirety and is not under consideration by another journal. All study participants provided informed consent, and the study design was approved by the appropriate ethics review board. We have read and understood your journal’s policies, and we believe that neither the manuscript nor the study violates any of these. There are no conflicts of interest to declare.

Thank you for your consideration. I look forward to hearing from you.

Sincerely,

Huasong Zeng

Department of Allergy, Immunology and Rheumatology,

Guangzhou Women and Children’s Medical Center,

Guangzhou Medical University,

Floor 11, Renmin Middle Road, Guangzhou, Guangdong, 510120, China

Tel: +86-020-81330667

Fax: +86-020-81330667

Email: zenghuasong@gwcmc.org
